# The All of Us Research Program’s Social Media Outreach to Underrepresented Populations: Mixed Methods Analysis

**DOI:** 10.2196/63793

**Published:** 2025-08-22

**Authors:** Dennis J Zhang, Michael G Bentz, Janet K Shim, Sandra S-J Lee

**Affiliations:** 1 Department of Biological Sciences, Columbia University, 1212 Amsterdam Ave, New York, NY, 10027, United States; 2 Department of Medical Humanities and Ethics, Columbia University, New York, NY, United States; 3 Department of Social & Behavioral Sciences, University of California, San Francisco, San Francisco, CA, United States

**Keywords:** precision medicine, underrepresented in biomedical research, research recruitment, social media, digital outreach, solidarity, data disparities, health disparities

## Abstract

**Background:**

The All of Us Research Program (AoURP) is a prominent precision medicine research initiative committed to diverse participation. The program harnesses digital outreach as a key strategy for recruiting and retaining underrepresented populations, using language that sometimes invokes notions of solidarity. This targeted recruitment of underrepresented groups and potential use of solidaristic language raise concerns about how participation will manifest tangible benefits for these populations and whether institutions assume responsibility for addressing past and present research harms.

**Objective:**

This study examines how the AoURP conceptualizes “diversity” in its social media outreach and how this implementation aligns with the program’s stated goals. Specifically, we perform a mixed methods analysis to descriptively capture (1) which underrepresented populations are targeted by the AoURP’s social media and (2) how solidaristic messaging is used, if at all, in these calls for participation.

**Methods:**

AoURP social media posts (n=380) from a 6-month period in 2020‐2021 were coded to identify visual depictions and explicit mentions of any “underrepresented in biomedical research” (UBR) categories officially targeted by the program. To then characterize UBR-specific appeals, we performed a thematic analysis of UBR-targeted posts, using a coding scheme that identified unsolidaristic language (ie, appeals to individual benefits) and solidaristic language (ie, appeals to benefitting others, attaining shared goals, and addressing injustices).

**Results:**

Among the 10 UBR categories officially recognized by the AoURP, “Race and Ethnicity” (187/380, 49% of posts) and “Age” (71/380, 19%) were the most frequently emphasized, while each of the other remaining categories was rarely invoked (<4/380, 1%). The thematic analysis further identified calls to participate that spanned receiving genetic results (ie, individual benefits), uncovering family and community disease predispositions (ie, benefitting others), improving the future of health (ie, achieving shared goals), and addressing data and health disparities (ie, resolving injustices).

**Conclusions:**

In addition to highlighting UBR categories that are more and less emphasized in the AoURP’s social media outreach, we also find that the program’s messaging indeed resembles a solidaristic appeal to participate. Drawing upon the existing literature on solidarity, we leverage conceptualizations of solidarity as a shared practice grounded in mutuality and bidirectionality to question the AoURP’s appeals when institutions fail to fully reciprocate this solidarity. Specifically, we raise concerns about (1) unclear links between participation and addressing health disparities, (2) incomplete acknowledgment of institutions’ role in data disparities, and (3) the use of empowerment rhetoric that diverts the onus for correcting these disparities onto participants. Finally, we consider the implications of these issues for future outreach efforts.

## Introduction

### The All of Us Research Program

The All of Us Research Program (AoURP) is a notable case study for exploring the conceptualization of “diversity” in precision medicine research. The program is one of the largest biomedical research initiatives in US history, aimed at accelerating precision medicine by longitudinally collecting varied data types over a 10-year period from a cohort of 1 million or more Americans [1,2] that is intended to “broadly reflect the diversity of the US” [3]. As of February 2024, the AoURP has already enrolled more than 700,000 people [4]. This core commitment to “setting the stage for more diverse participation in precision medicine” stems from the recognition that “not all groups have benefited equally from research” [5]. In 2020, the program delineated criteria for “underrepresented in biomedical research” (UBR) [5], which it has since used to measure its success in recruiting a diverse cohort [6].

Digital outreach functions as a major component of the AoURP’s efforts to enroll underrepresented participants. For instance, as of May 2025, the AoURP’s 4 main social media accounts across X (formerly Twitter at the time of data collection and thus referred to as such throughout this study), Facebook, Instagram, and YouTube garner a collective audience of more than 200,000 followers. Moreover, during the COVID-19 pandemic, the program’s inability to rely on in-person engagement and achieve enrollment targets motivated it to ramp up its social media outreach campaigns [6]. The AoURP reports that “these campaigns experimented with varying value propositions for research participation, including the return of personal health-related information and altruism,” which the AoURP credits with a steady increase in enrollment activities [6]. However, despite the reliance of programs such as the AoURP on social media for recruitment, Gelinas et al [7] and Hokke et al [8] have pointed out the general dearth of regulatory guidance on such outreach.

Leveraging the AoURP’s social media presence as a rich locus for exploring how the program conceptualizes and implements “diversity,” this study aims to begin addressing this gap with a mixed methods analysis by (1) gauging which populations are being appealed to and (2) exploring what kind of language the AoURP uses to justify these calls for participation. Interestingly, before analyzing the social media dataset, we observed elsewhere in the AoURP’s digital outreach, such as on its official website [1], that the AoURP calls for participants to join and share health information and samples to “help improve the health of your communities and future generations,” thereby using collectivist language that invokes notions of solidarity. Moreover, other scholars such as Neuhaus [9] have similarly drawn upon the framework of solidarity to understand the appeal to participate in such research programs. Thus, we explored the AoURP’s calls to participate in our social media dataset with a specific interest in solidaristic language.

### Background on Solidarity

Prainsack and Buyx [10] were the first to take a systematic approach to compiling, formulating, and clarifying the concept of solidarity. In essence, Prainsack and Buyx conceptualized solidarity as “shared practices reflecting a collective commitment to carry ‘costs’ (financial, social, emotional, or otherwise) to assist others.” These acts are often preceded by the recognition of sameness with another person or group. Furthermore, Prainsack and Buyx emphasize that solidarity, unlike empathy and similar concepts, is “understood here as a practice and not merely as an inner sentiment.” This property makes solidarity a particularly suitable framework for understanding the explicit calls to action characteristic of the AoURP’s outreach (eg, “#JoinAllofUs”).

Other scholars have since put forth alternative models of solidarity. Arguing that sameness-based models of solidarity may exclude those seen as different, O’Neill [11] has devised a second notion of “conjoint solidarity” that is more inclusive of diverse health care stakeholders. Specifically, O’Neill envisions solidarity as based around a shared goal (eg, “the shared goal of improved health care outcomes”) rather than similarity. Finally, Dawson and Jennings [12] have conceived a third notion of solidarity that instead centers around injustices. This reconfigured practice of solidarity is best captured by the fundamental idea of “standing up beside,” where solidarity requires a public action that is a positive identification with another and is oriented toward improving or correcting past or present disadvantage.

These frameworks understand solidarity as a practice built around mutuality and bidirectionality that can be shared with and expected from organizations [11,12]. Notably, other scholars have recently pointed out and clarified the unmet obligations of the AoURP to its participants. For instance, Jabloner and Walker [13] argue that “one cannot simply assume that more diverse datasets will automatically lead to better health care for the underserved” and that initiatives such as the AoURP must make explicit how research participation will translate into tangible benefits for the underrepresented, or risk solely benefiting those in power without meaningfully advancing health equity. Additionally, Tsosie et al [14] have further elucidated the historical and ongoing research exploitation of underrepresented populations, raising questions about the extent to which institutions claim accountability for this harm. In light of these arguments, we conducted a mixed methods analysis of whether and how the AoURP upholds its solidaristic obligations to its participants by first identifying (1) who the AoURP’s social media campaign appeals to and (2) what is embedded in its call to participate.

## Methods

### Data Collection

We first accessed and captured screenshots of all posts across the AoURP’s 4 main social media accounts (“@AllofUsResearch” on Twitter, “All of Us Research” on Facebook, “@allofusresearch” on Instagram, and “All of Us Research Program” on YouTube) that were published between September 10, 2020, and March 10, 2021. Data collection was bounded at these dates to capture a sizable sample as the AoURP ramped up its social media campaign during the COVID-19 pandemic [5]. In total, we captured 449 posts: 265 from Twitter, 140 from Facebook, 40 from Instagram, and 4 from YouTube.

**Figure 1.**
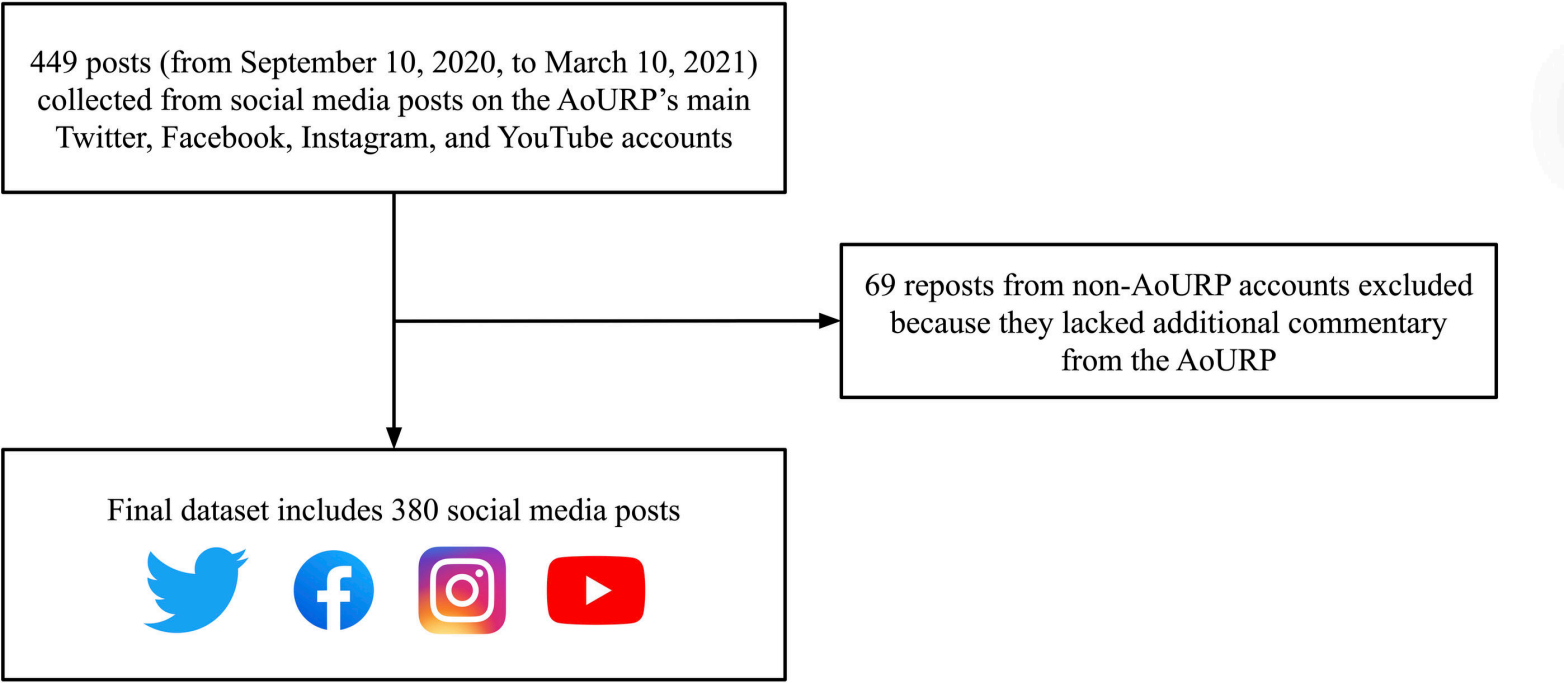
Schematic of inclusion and exclusion criteria. AoURP: All of Us Research Program.

Because of our intent to analyze the AoURP’s outreach, we chose to focus our sample on posts that included original content created by the program. We therefore excluded 69 posts that were reposts from other non-AoURP social media accounts and lacked any additional commentary (eg, an added caption) from the main AoURP account. After removing posts based on this exclusion criterion, our final dataset consisted of 380 posts: 199 from Twitter, 137 from Facebook, 40 from Instagram, and 4 from YouTube (Figure 1).

The captions of all posts were transcribed. For posts in Spanish (which was the only language other than English used by the AoURP), if the post platform did not have its own autotranslate feature (eg, as offered by Facebook), we translated each post via Google Translate and added this translation to the caption transcription. Previous work has argued that Google Translate is a useful tool for reducing language bias and is sufficiently accurate in translating Spanish to English for research contexts [15]. Regardless, each translation was manually inspected for accuracy by 1 author (DJZ), who can proficiently read Spanish.

### Data Analysis

#### Aim 1: Which Underrepresented Populations Does the AoURP Appeal to?

Within our dataset, we sought to first code posts based on whether they targeted underrepresented populations and, if so, which populations. In the “*All of Us* Guide for Diversity and Inclusion” [5] and on its official website [16], the AoURP outlines 10 UBR categories and numerous subcategories used to enroll participants. We created codes and subcodes that corresponded to each UBR category and subcategory. As asserted by other scholars, image and textual references on social media are not accidental but instead strategically selected to promote research participation by making outreach materials more culturally appropriate [17,18]. Thus, we treated visual depictions of and textual references to any AoURP-defined UBR population as an appeal to that population and coded that post accordingly. Indeed, messaging found in AoURP posts from our dataset reaffirmed our methodological approach. In one of the AoURP’s longer-form video testimonial posts, an African American narrator explicitly acknowledges how visual representation can impel participation: “I want to be the face that says to others who look like me, ‘Please participate and be counted.’”

Based on our examination of the dataset, we also chose to expand UBR codes beyond the categories used by the AoURP. We added a “nonspecific” code to capture any appeals that explicitly target UBR populations without referencing any 1 category in particular (eg, by mentioning “groups that are underrepresented in biomedical research”). Under the “Race and Ethnicity” code, we also included a supplemental “Other” subcode for posts explicitly invoking a non-White racial or ethnic identity (eg, “Native American”) that lies outside of the 5 demographics specified by the AoURP (ie, “Asian,” “Black, African or African American,” “Hispanic, Spanish, or Latino,” “Native Hawaiian or Pacific Islander,” and “Middle Eastern or North African”). Finally, we added an “Ambiguous” subcode for posts where assigning a “Race and Ethnicity” subcode proved difficult (see the section “Limitations” for a discussion on the challenges of visually assigning racial and ethnic identities). We intentionally supplemented our visual annotation strategy with a text-based approach, in which UBR categories needed to be explicitly invoked, to further improve our methodology’s rigor. The final codebook that guided our analysis is shown in Table 1.

**Table 1.** “Underrepresented in biomedical research” (UBR) codebook used to classify the dataset.

UBR code and subcodes^a^	Description
“Race and Ethnicity” (ie, “Asian,” “Black, African or African American,” “Hispanic, Spanish, or Latino,” “Native Hawaiian or Pacific Islander,” “Middle Eastern or North African,” *Other, Ambiguous*)	“Individuals who identify as other than White and non-Hispanic”
“Access to Care”	“Individuals who have not had a needed medical visit in the past 12 months or cannot easily obtain or pay for medical care as needed”
“Age” (ie, “Children 17 or younger,” “Adults 65 or older”)	“Children 17 or younger and adults 65 or older”
“Annual Household Income”	“Individuals with household incomes equal to or below 200% of the Federal Poverty Level”
“Disability”	“Individuals with either a physical or cognitive disability”
“Educational Attainment”	“Individuals with less than a high school degree or equivalent”
“Gender Identity”	“Individuals who identify as gender variant, non-binary, transgender, or something else”
“Geography”	“Individuals who reside in rural and non-metropolitan areas”
“Sex Assigned at Birth”	“Individuals who are neither male nor female (ie, intersex)”
“Sexual Orientation”	“Individuals who identify as asexual, bisexual, gay or lesbian or something else”
*Nonspecific*	*Individuals who are broadly categorized as underrepresented but whose specific group is unnamed* (*eg, “groups that are underrepresented in biomedical research”*)

a Codes and subcodes with quotation marks came verbatim from the All of Us Research Program [5,16], whereas codes and subcodes in italics came from the authors. Only “Race and Ethnicity” and “Age” have subcodes listed because these were the only codes with related subcodes assigned to any posts.

#### Aim 2: What Appeals Is the AoURP Using to Call the Underrepresented to Participate?

After identifying UBR-targeted posts, we sought to characterize how exactly the AoURP was calling these underrepresented populations to participate. Using guidelines set forth by Braun and Clarke [19], we performed a thematic analysis of such appeals involving the following stages: familiarization with the data, generating initial codes, searching for themes, reviewing themes, defining and naming themes, and producing the report. Given our specific interest in solidarity [10-12], we methodologically implemented this focus while developing our coding scheme by looking for instances where related concepts were invoked. This led us to code language that was seemingly either unsolidaristic (ie, appeals to individual benefits) or solidaristic (ie, appeals to benefitting others [10], achieving shared goals [11], and resolving injustices [12]). Overall, DJZ led the initial analysis, and through iterative discussions of codes and data with other authors (JKS and SS-JL), we refined the themes presented in the “Results” section.

### Ethical Considerations

This study draws on publicly available data from X (formerly Twitter), Facebook, Instagram, and YouTube, and does not include any interaction with human subjects. No identifying information of individuals who posted content was collected, analyzed, or reported. All data analyzed were deidentified and publicly accessible at the time of collection. In accordance with current ethical guidelines for research using publicly available online content, this study was determined to be exempt from institutional ethics review.

## Results

### Which Underrepresented Populations Does the AoURP Appeal to?

In our dataset, we observed a striking emphasis on posts targeting “Race and Ethnicity” UBR populations (187/380, 49% of all posts), followed by “Age” (71/380, 19%) and “Nonspecific” invocations of underrepresented populations (19/380, 5%) (Table 2).

**Table 2.** Textual and visual references to the “underrepresented in biomedical research” (UBR) codebook by the number of posts (n) and proportion (%) relative to the total dataset (n=380).

UBR code and subcodes^a^	Textual references	Visual references	Either reference type
*Any*, n (%)	88 (23)	172 (45)	209 (55)
“Race and Ethnicity,” n (%)^b^	65 (17)	159 (42)	187 (49)
“Asian”	2 (1)	29 (8)	30 (8)
“Black, African or African American”	21 (6)	106 (28)	113 (30)
“Hispanic, Spanish, or Latino”	38 (10)	49 (13)	73 (19)
*Other*	1 (0)	—^c^	1 (0)
*Ambiguous*	—	5 (1)	5 (1)
“Age,” n (%)	2 (1)	69 (18)	71 (19)
“Children 17 or younger”	—	42 (11)	42 (11)
“Adults 65 or older”	1 (0)	32 (8)	33 (9)
*Nonspecific*, n (%)	19 (5)	—	19 (5)
“Gender Identity,” n (%)	3 (1)	—	3 (1)
“Sex Assigned at Birth,” n (%)	3 (1)	—	3 (1)
“Geography,” n (%)	2 (1)	—	2 (1)
“Sexual Orientation,” n (%)	2 (1)	—	2 (1)
“Access to Care,” n (%)	—	—	—
“Annual Household Income,” n (%)	—	—	—
“Disability,” n (%)	—	—	—
“Educational Attainment,” n (%)	—	—	—

a Codes and subcodes with quotation marks came verbatim from the All of Us Research Program [5,16], whereas codes and subcodes in italics came from the authors. Only “Race and Ethnicity” and “Age” have subcodes listed because these were the only codes with related subcodes assigned to any posts.

b The “Race and Ethnicity” code could be assigned only once to a particular post, even if multiple subcodes within “Race and Ethnicity” were assigned to that same post. The same was true for the “Age” code.

c Absence of posts.

Within “Race and Ethnicity,” the most commonly applied subcodes were “Black, African or African American” (113/380, 30% of all posts); “Hispanic, Spanish, or Latino” (73/380, 19%); and “Asian” (30/380, 8%). After data collection, we observed that the time period of our dataset (see the “Methods” section) included Black History Month and Hispanic Heritage Month but did not include Asian American and Pacific Islander Heritage Month. To assess the impact of these heritage months on our dataset, we excluded all heritage month posts (n=131) and found that this did not substantially change the relative distribution of appeals across these “Race and Ethnicity” subcodes: “Black, African or African American” (66/249, 27% of all remaining posts); “Hispanic, Spanish, or Latino” (44/249, 18%); and “Asian” (23/249, 9%).

In contrast, we observed a simultaneous dearth of textual and visual references to the remaining UBR codes, namely, “Gender Identity” (3/380, 1% of all posts), “Sex Assigned at Birth” (3/380, 1%), “Geography” (2/380, 1%), “Sexual Orientation” (2/380, 1%), “Access to Care” (0/380, 0%), “Annual Household Income” (0/380, 0%), “Disability” (0/380, 0%), and “Educational Attainment” (0/380, 0%).

Despite this difference in emphasis, the AoURP deems all of its 10 identified UBR categories pivotal for the program’s enrollment goals and that research must account for a broader range of demographics [5]. The AoURP’s apparent prioritization of the “Race and Ethnicity” category may reflect disproportionately greater decreases in enrollment during the pandemic from “Black/African American” and “Hispanic/Latino/Spanish” populations [6]. However, we did not observe any parallel social media outreach efforts to explicitly engage underrepresented income and educational attainment groups, which also experienced disproportionate enrollment decreases during the pandemic [6].

### What Appeals Is the AoURP Using to Call the Underrepresented to Participate?

In posts categorized as targeting the underrepresented (209/380, 55% of all posts), we identified calls to participate that spanned diverse appeals to individual benefits, serving one’s family and community, achieving shared goals, and resolving injustices.

#### Theme 1: Receiving Genetic Results: Participate to Gain Individual Benefits

A total of 37 posts in our dataset included appeals to the return of genetic results as a means of emphasizing individual benefits from participation. According to the AoURP, one of their core commitments and novelties as a research program is “ensuring that participants have access to their own information,” given that many report “a strong desire to understand what their DNA can tell them” [20]. This commitment gave rise to the return of genetic results that include “non-health-related” traits (eg, lactose intolerance, caffeine sensitivity, etc) and genetic ancestry. Notably, during our sampling time frame, the AoURP had not yet begun returning “health-related” traits (eg, genetic risk for certain diseases, pharmacogenetics, etc). The posts related to the return of results that were captured in our dataset mainly appealed to individual excitement for learning more about oneself:

*One of the most exciting parts of participating in our program is the option to receive your #DNA results! We’ve begun to analyze participant DNA for info+give participants their results, if they choose to receive them*.

[Twitter post]

*Did you know some people feel sick when they have milk and other dairy foods? Lactose intolerance is a #DNA trait. We’re analyzing and returning genetic ancestry and trait results to some of our participants*.

[Facebook post]

The majority of such return of results posts targeted underrepresented groups (23/37, 62%) and, more specifically, racial and ethnic minorities (22/37, 59%) with an emphasis on Hispanic (14/37, 38%) and African Americans (16/37, 43%):

*We have already started sending #genetic results to All of Us participants who donated their blood or saliva samples to science. Participants can choose to receive information about their #ancestors and genetic traits*.

[Twitter post, originally in Spanish]

#### Theme 2: Uncovering Disease Predispositions: Participate to Benefit One’s Family and Community

Explicit appeals to family health history extend individual benefits to further emphasize benefits to those related or similar to the individual. These posts, while relatively infrequent (n=8), emphasize how sharing family health history with researchers and participating in the AoURP can help shed light on personal and family disease risk:

*Happy Family Health History Month! Did you know your family health history can help researchers better understand diseases and conditions that may impact you and your family?*

[Facebook post]

Other posts further emphasize disease predispositions that are more broadly associated with one’s racial or ethnic group:

*Q: Does COVID-19 only affect people from a certain racial or ethnic group? A: Regardless of your race or ethnicity, COVID-19 can affect anyone. It’s been more significantly impacting the Black, Latinx, and Native American populations*.

[Facebook post]

This approach is further evident in a handful of longer-form posts targeting African and Hispanic Americans. These posts do not solely trace such predispositions to one’s genetics; instead, they invoke group-specific lifestyle and environmental factors to contextualize health risks:

*Hear [narrator] discuss family health history+how gathering more data can help improve #healthresearch in Black American communities … [narrator:] I definitely think that there’s health conditions particularly as an African American female, that my family history has predisposed me to. These are personal experiences that I’ve seen and experienced growing up, whether it’s relatives now that are suffering from various chronic conditions, and if our African American ancestors and our families knew more of the information, to help them lead a healthier lifestyle, [it] really would help us live longer healthier lives. #JoinAllofUs.*

[Twitter post]

*[Narrator] talks about how our genes and environment may impact our health. … [narrator:] I interpret for patients in the medical center. I also do CPS (Child Protective Services) cases, schools, community services. I do work with a lot of immigrants. Mostly Central America and Mexico. People are developing diabetes, obesity, allergies. There is a whole range of diseases. A lot of it has to do not only with the environment but also eating healthy. It’s very expensive in the States.*

[Facebook post]

Elsewhere, these group-specific appeals take the form of “disease awareness” posts, which are often timed with national disease awareness efforts:

*Heart disease is the leading cause of death for Hispanics/Latinos in the US #JoinAllofUs.*

[Twitter post, originally in Spanish]

*#DidYouKnow that diabetes affects millions of Hispanic families?*

[Twitter post, originally in Spanish]

*As we bring awareness to Sickle Cell Disease, #DYK it occurs amongst 1 out of every 365 Black or African-American births? ... #SickleCellAwarenessMonth.*

[Twitter post]

Indeed, disease-related posts frequently include textual and visual references to underrepresented racial and ethnic groups (Table 3).

**Table 3.** The most frequently mentioned diseases in the dataset and their associated references to specific racial and ethnic groups^a^.

Disease focus	Total posts, n	“Black, African or African American,” n (%)	“Hispanic, Spanish, or Latino,” n (%)	“Asian,” n (%)
COVID-19	129	37 (29)	16 (12)	3 (2)
Cardiovascular disease	8	4 (50)	2 (25)	—^b^
Diabetes	8	3 (38)	6 (75)	—
Sickle cell disease	3	2 (67)	—	—

a For each disease, the total number of posts (n) is depicted, alongside the number of posts with textual or visual references to each “Race and Ethnicity” subcategory. Proportions (%) are relative to the total number of posts per disease.

b Absence of posts.

#### Theme 3: Improving the Health of Future Generations: Participate to Achieve Shared Goals

Among the most common appeals in the dataset are calls to improve the health of future generations (n=45). Similar to participating for one’s family or community, this type of appeal emphasizes a shared sense of responsibility. For example, posts assert that “By participating, you and your community could have an impact on the health of future generations” and that this “healthier future [is] for all.”

Of these posts, the vast majority targeted underrepresented groups (41/45, 91%) and, specifically, racial and ethnic minorities (38/45, 84%). We also observed a notable emphasis on Hispanic (23/45, 51%) and African American (22/45, 49%) populations, which often coincided with Hispanic Heritage Month and Black History Month. These posts invoke the language of collective responsibility to encourage the associated racial and ethnic groups to participate, for example, “This Black History Month we honor the past, to help shape the future. #JoinAllofUs to help drive research forward.”

On how exactly a healthier future will be achieved, the AoURP asserts that diverse participation will increase scientific discovery potential, enabling scientists to drive, improve, and speed up research (n=40); learn more about health (n=26); find new ways to stay healthy (n=22); better understand diseases (n=17); answer important health questions (n=13); and identify patterns such as how biology and background can affect health (n=6):

*All of Us is committed to helping improve health research by including people from all backgrounds. You can sign up to help drive research forward at JoinAllofUs.org*.

[Twitter post]

*As an All of Us Research Program participant, you are already helping in more ways than you know. You are part of a group of 350,000+ participants from all 50 states. With participation from so many people, we have the power to help researchers answer important health questions*.

[Facebook post]

#### Theme 4: Data and Health Disparities: Participate to Help Address Injustices

The AoURP’s widespread appeals to underrepresented groups (n=209) implicitly draw upon the injustice of data disparities in research participation as a collective impetus for participation. In some instances, these data disparities are linked to health disparities. For example, in longer-form posts, the narrator asserts that by including populations who have been “historically left out” in the AoURP, researchers can “help end health disparities”:

*Hear [narrator] share his experience with #COVID19 and why he thinks researchers can help end health disparities. … [narrator:] I sit back and I think to myself, so these same communities that we’re reaching out to and asking to participate in this research program because they have been historically left out and marginalized are taking the brunt of this crisis*.

[Facebook post]

In general, such mentions of health disparities (n=6) are relatively scarce in the dataset, and when they do occur, they most often reference COVID-19–related disparities (n=3). Notably, due to a large overlap between our entire dataset and COVID-19–invoking posts, it was difficult to perform a direct analysis of the latter that is fundamentally distinct from what we have already presented. That said, outside of COVID-19, the other most commonly referenced diseases in our dataset (Table 3), namely, cardiovascular disease, diabetes, and sickle cell disease, are all known to have long-standing health disparities, even if these disparities are not always explicitly mentioned in posts [21-23].

Curiously, when such health disparities *are* explicitly invoked, their precise link to data disparities is left unarticulated. For instance, the AoURP implores the underrepresented to participate in order to advance understanding of risk factors without fully explaining how that will translate tangibly toward addressing health disparities:

*#JoinAllofUs and help scientists understand risk factors and discover how to address health disparities in your community*.

[Twitter post, originally in Spanish]

A similar lack of explanation is also seen in occasional AoURP social media posts promoting the NIH UNITE initiative (n=3), which aimed to address structural racism and promote racial equity and inclusion within the biomedical research community. These posts direct audiences to external links that feature a longer-form statement by the AoURP CEO on the UNITE initiative. This statement acknowledges how the COVID-19 pandemic has “exposed long-standing differences in health care, access, and outcomes” and then harnesses these health disparities as a justification for addressing data disparities, without articulating how exactly the latter will help resolve the former:

*The COVID-19 pandemic, which has claimed more than 500,000 lives in the United States, has again exposed long-standing differences in health care, access, and outcomes. These realities are stark reminders about why we must always strive to do more to ensure everyone is represented in research*.

[Statement by the AoURP CEO, externally linked to by Facebook post]

Moreover, in light of this tenuous link between data and health disparities, we were curious about how the AoURP specifically calls UBR groups to address data disparities. In one especially salient video testimonial, an African American narrator states:

*The biggest benefit of the All of Us Research Program is that, for once, a group of researchers have actually specifically sought out the underrepresented groups in society to make sure we’re represented in research*.

[Facebook post]

This surface-level account (that the exclusion of underrepresented groups is or was solely due to their being “left out” by researchers) contrasts with other instances, outside of our dataset, where the AoURP acknowledges a more comprehensive range of causes for such disparities, that is, active research harms and exploitation. For instance, in the aforementioned external statement on the UNITE initiative:

*We recognize that many individuals and communities have been mistreated and abused by or left out of research. These transgressions have contributed to the ongoing health disparities and inequities that continue to harm so many in this country*.

[Statement by the AoURP CEO, externally linked to by Facebook post]

In light of the AoURP’s inconsistent acknowledgment of such institutional harms, it is curious how responsibility is sometimes redirected onto underrepresented participants. Later in the aforementioned video testimonial by an African American narrator, the narrator responds to researchers’ interest in African Americans:

*And since that call has been placed out there, that people are interested in African Americans and learning about disease process, disease progression, I think a part of that response as an African American is to stand up and say that I’m willing to participate. I signed up for the All of Us Research Program because I represent a group that has historically been underrepresented in research and I want to be counted. And I want to be the face that says to others who look like me, Please participate and be counted.*

[Facebook post]

Interestingly, this shifting of responsibility onto participants (“part of that response as an African American is to stand up and say that I’m willing to participate”) echoes the AoURP’s broader use of empowerment rhetoric throughout the dataset, which underscores participants’ “power” to enact tangible change:

*You have the power to help drive research with our new research plans and program activities*.

[Facebook post]

*[AoURP CEO:] By learning from the past, we all have the power and responsibility to help change the future of health in our communities. If you join @AllofUsResearch, you may help #researchers find new ways for all of us to stay healthy*.

[Twitter post]

Collectively, the range of these textual and visual references to particular UBR populations, as well as the diverse themes embedded in the associated messaging, underscores the multifaceted ways that the AoURP calls for the underrepresented to participate.

## Discussion

### Principal Results

In analyzing AoURP social media posts, this mixed methods study identified (1) who among the underrepresented is being called to participate and (2) what messaging is being leveraged in that call. While the AoURP deems all of its 10 identified UBR categories to be pivotal for the program’s enrollment goals and that research must account for a wider range of demographics [5], we found a primary emphasis on the UBR categories of “Race and Ethnicity” and “Age” that contrasts with a lack of references to other categories. Moreover, the AoURP rhetorically incentivizes participation from individuals in these underrepresented groups by framing it as an opportunity to receive genetic results, uncover disease predispositions to benefit their families and communities, advance the shared goal of improving the future of health, and even address injustices such as data and health disparities.

### Comparison With Prior Work

While some of these aforementioned appeals are more individualistic and seemingly less solidaristic (ie, receiving genetic results), the remaining appeals align closely with existing models of solidarity and suggest that the AoURP’s outreach can indeed be understood as a solidaristic call to participate.

A hallmark of Prainsack and Buyx’s [10] framework is that “solidaristic acts are preceded by the recognition of sameness with another person or group.” This focus on sameness is present in the AoURP’s appeals to the family- and community-specific benefits of participation. Notably, these posts often leverage audience members’ connection to communities of shared race or ethnicity by visually depicting and explicitly invoking such groups. Additionally, the embedded message that participation can lead to new knowledge about disease predispositions that would apply to others of the same race or ethnicity implicitly invokes ideas about presumed biological similarity among members of the same racial or ethnic group. Given this, it is worth considering whether more explicit references to *social* grounds of affinity (eg, in terms of history, social experience, etc) would help avoid the misstep of inferring biological sameness or difference from race and ethnicity.

Moreover, aligned with O’Neill’s [11] conceptualization of “conjoint solidarity” that revolves around a shared goal rather than perceived similarity with others, one of the most prevalent appeals in our dataset is the notion that prospective participants can work together to improve the health of future generations. As envisioned by O’Neill, this repeated messaging is often framed by the AoURP in an identity-agnostic manner that appears inclusive to all prospective participants.

Finally, in agreement with Dawson and Jennings’ [12] model of solidarity as the idea of “standing up beside” (ie, a “public action” oriented toward addressing injustice), the act of participating in the AoURP can indeed be understood to be “public” as individuals who enroll in the program may share their participation with others. As an especially visible example, video testimonials narrated by AoURP participant ambassadors, that is, participants designated to act as representatives for the research program, are present throughout the dataset. As we describe, in one such testimonial, an AoURP participant ambassador pronounces her identity as an African American, frames her participation as a response to this population’s historical underrepresentation in research, and further implores other African Americans to similarly join. Here, the ambassador explicitly invokes the solidaristic call to “stand up” beside the African American community, positively identifying with them and participating in direct action perceived to help resolve past injustice.

### Broader Implications

While the AoURP uses solidaristic language to call for participation, we posit that the program’s social media outreach may not realize the full definition of solidarity as a practice built around mutuality and bidirectionality that can be shared with and expected from biomedical institutions [11,12]. In particular, we raise the following critical issues and consequences for future outreach efforts.

#### Issue 1: An Unclear Causal Relationship Between Participation and Resolving Health Disparities

Our data demonstrate that the AoURP social media campaign harnesses health disparities as an impetus for participation, often without explaining how data equity will resolve such disparities in the first place. Jabloner and Walker [13] argue that the flawed notion that “benefit for underserved populations will flow directly from more diverse datasets and associated biomechanistic research tailored to ‘minority populations’” must be urgently revised, or risk a form of “predatory inclusion” that solely benefits those in power without progressing toward health equity [24]. Indeed, acts of solidarity (ie, participation in the AoURP) may be directed toward resolving past or present injustice [12], but we posit that a failure to clearly show how the act of participation will reduce health disparities risks making an unfulfilled promise to participants and, therefore, may fall short of realizing the full definition of mutualistic and bidirectional solidarity as described by O’Neill [11] and Dawson and Jennings [12].

#### Issue 2: Insufficient Elaboration of Institutions’ Active Role in Driving Data Disparities

Along a similar vein, the AoURP does not consistently articulate the full historical role and culpability of research institutions in creating the very data disparities the program aims to directly address. In our dataset, underrepresentation is often depicted simply and almost straightforwardly as the existing reality or sometimes framed as a sole consequence of researchers being less attentive or committed to inclusion. This passive narrative—that the underrepresented have been simply overlooked—fails to acknowledge a history of research institutions actively excluding some populations and subjecting them to harm and exploitation. The AoURP does not directly acknowledge this history in our dataset outside of a handful of social media posts that externally link to a statement on the UNITE initiative. As among the most infamous case studies, the infliction of research harms upon African Americans in the Tuskegee Syphilis Study was an institutional betrayal of trust that has become culturally embedded and still drives suspicion from prospective research participants [25]. Along a similar vein, Tsosie et al [14] critique a vicious cycle of past and present research exploitation of Indigenous populations, who disengage from research due to the harm it often imposes, yet are told that they will miss out on the benefits of precision health if they do not engage, with no attention paid to preventing future harm or addressing underlying health inequities. Failing to acknowledge these histories may amount to another instance of not reciprocating UBR participants’ solidaristic acts of participation. Therefore, thinking about frameworks of solidarity in this context helps reveal the gap between the enrollment practices we analyze and calls to participate in precision medicine research initiatives such as the AoURP, and whether and how they generate knowledge that will materially address health disparities.

#### Issue 3: Deflection of Responsibility for Correcting These Disparities Onto Participants by Using Empowerment Rhetoric

Indeed, the duty of ameliorating ongoing disparities ironically seems to fall less on research institutions and more on the underrepresented populations themselves. As we describe, in one video testimonial example from our dataset, an African American participant implores other African Americans to capitalize on the opportunity of finally being seen. Here, the rhetoric of empowerment, where participation is framed as a reclamation of power (“stand up,” “be counted”), suggests that individual assumption of responsibility can manifest real change. Such language features prominently throughout the data, often reinforcing the notion that individuals have the power to drive research. However, McLaughlin [26] points out that such empowerment rhetoric has historically diverted focus from the “external world of political, economic or structural change” to the “inner world” of individuals as the unit from which both problem and solution emerge. Indeed, it should not be overlooked how the AoURP’s emphasis on the power of individual participation to correct injustices in data diversity and representation (a claim that many would argue is itself dubious) obscures institutional responsibility for similarly enacting change, which, again, may amount to an incomplete realization of the reciprocal nature of solidarity [11,12].

#### Consequences for Future Outreach

The AoURP presents a valuable case study for understanding the complexities of participant recruitment in the context of massive biomedical research initiatives. The program must balance the competing demands of efficiently enrolling large numbers of participants while also emphasizing the recruitment of those who are underrepresented. This tension leads to a fine line of promotional messaging that must attract hundreds of thousands of participants, especially those from targeted groups, while refraining from overpromising. Even though the *All of Us* Institutional Review Board may not be the only possible source of guardrails against imbalanced messaging, it is responsible for reviewing and approving numerous participant-facing materials [27]. As such, the *All of Us* Institutional Review Board likely remains the primary governance structure through which this messaging should be regulated. Moving forward, it remains critical to convey the purpose of these biodata repositories to lay audiences in a manner that is accessible [28] and yet avoids making implied promises that lack tangible paths to being achieved.

Indeed, rather than solely modifying its messaging and risking rhetorical overreach, programs such as the AoURP can consider implementing services and arrangements that provide tangible benefits for underrepresented participants. For example, Jabloner and Walker [13] propose various solutions that range from data donors receiving health care in exchange for participation to benefit-sharing, where donors receive financial benefits from the revenue generated by their data. The recent success of a benefit-sharing agreement between Variant Bio and Indigenous peoples exemplifies that such solutions may be a practical step that enables the AoURP to achieve its engagement goals while upholding the full obligations of its solidaristic call to participate [29].

### Limitations

While our efforts to code visual references to underrepresented groups in the AoURP’s social media posts were motivated by our desire to capture how images and, more specifically, representation in images can enhance the targeting of appeals to participate [17,18], the process of identifying such references is not without limitations. As recommended by Golder et al [30], we acknowledge both systematic and individual biases that can influence the reproducibility of our results. Multiple studies have identified such biases when classifying individuals into racial and ethnic groups based on photographs [31,32]. These biases may contribute to the apparent absence of posts categorized as appealing to “Race and Ethnicity” subcategories outside of “Asian,” “Black, African or African American,” and “Hispanic, Spanish, or Latino” [5]. That said, considering textual references alone, where specific racial and ethnic groups were explicitly named, shows that these 3 “Race and Ethnicity” subcategories are still the most commonly targeted in our dataset (Table 2). Additionally, as of January 2024, the AoURP has created outreach materials that specifically and exclusively target these 3 subcategories, for example, in making “Asian American,” “African American,” and “Hispanic American” iterations of a generic recruitment brochure [33].

### Conclusions

Although social media is a critical resource for biomedical research programs to recruit and retain participants, there remains a dearth of literature exploring and guiding the messaging being promulgated through this medium. In response to this gap, this mixed methods study examines how one such program, the AoURP, harnesses social media to call for participation. Moreover, our intentional focus on the AoURP stems from its explicit emphasis on enrolling diverse participants, which may allow this study to broadly elucidate how similar precision medicine research programs conceptualize and implement “diversity.” Indeed, we draw attention to an emphasis on UBR categories of “Race and Ethnicity” and “Age” in contrast with a disproportionate lack of references to “Gender Identity,” “Sex Assigned at Birth,” “Geography,” “Sexual Orientation,” “Access to Care,” “Annual Household Income,” “Disability,” and “Educational Attainment.” Finally, this study both leverages and expands upon the framework of solidarity to characterize the justifications for participation put forth by the AoURP in targeting these UBR populations, thereby offering a starting point for the refinement of such messaging.

## Abbreviations

AoURP: All of Us Research Program
UBR: underrepresented in biomedical research
